# Deep Neural Network Approach for Pose, Illumination, and Occlusion Invariant Driver Emotion Detection

**DOI:** 10.3390/ijerph19042352

**Published:** 2022-02-18

**Authors:** Susrutha Babu Sukhavasi, Suparshya Babu Sukhavasi, Khaled Elleithy, Ahmed El-Sayed, Abdelrahman Elleithy

**Affiliations:** 1 Department of Computer Science and Engineering, University of Bridgeport, Bridgeport, CT 06604, USA; ssukhava@my.bridgeport.edu (S.B.S.); susukhav@my.bridgeport.edu (S.B.S.); aelsayed@bridgeport.edu (A.E.-S.); 2 Department of Computer Science, William Paterson University, Wayne, NJ 07470, USA; elleithya@wpunj.edu

**Keywords:** deep neural networks, advanced driver assistance systems (ADAS), face detection, K.L.T., MTCNN, facial expression recognition, driver emotion detection, DeepNet, machine learning

## Abstract

Monitoring drivers’ emotions is the key aspect of designing advanced driver assistance systems (ADAS) in intelligent vehicles. To ensure safety and track the possibility of vehicles’ road accidents, emotional monitoring will play a key role in justifying the mental status of the driver while driving the vehicle. However, the pose variations, illumination conditions, and occlusions are the factors that affect the detection of driver emotions from proper monitoring. To overcome these challenges, two novel approaches using machine learning methods and deep neural networks are proposed to monitor various drivers’ expressions in different pose variations, illuminations, and occlusions. We obtained the remarkable accuracy of 93.41%, 83.68%, 98.47%, and 98.18% for CK+, FER 2013, KDEF, and KMU-FED datasets, respectively, for the first approach and improved accuracy of 96.15%, 84.58%, 99.18%, and 99.09% for CK+, FER 2013, KDEF, and KMU-FED datasets respectively in the second approach, compared to the existing state-of-the-art methods.

## 1. Introduction

The current way of human living relies on intelligent vehicles developed with artificial intelligence. These smart vehicles make life easier for people who are busy in their daily lives. The lifestyle controls human actions in most situations in their daily routine. The most influenced situation of any human being that leads to severe damage to his life is the effect of emotions while vehicle driving on roads.

A driver’s attention will get distracted when they are in the emotional stage which will affect the alertness level and judging capability from normal conditions which are inadequate in safe driving. In total, 2.2% of fatalities are caused by vehicle crashes, according to annual road crash statistics [1], and 90% of accidents are causing due to human errors of the driver on roads. Studies proved that around 43% of crashes were avoided by co-passengers who alerted the drivers by observing their emotions instantly. As the emotion controls the mental status of the driver, it is essential to install an artificial intelligence system to assist the drivers in alerting them to be free from the emotion that influences driving behaviors and road safety. Therefore, the current generation of vehicles must include a function to alert the driver depending on their state of emotion. Several technical developments have been made in vehicles’ systems for a decade, which are accessible to the drivers inside the vehicle to track the driver’s emotions before causing accidents. These systems assist “ADAS” (advance driver assistance systems), which can help improve driver’s safety and provide enough potential for the driver to react early before road accidents.

Detection of human emotion from the camera-captured images is a reliable monitoring source for various safety and security applications. This can be achieved through facial expression recognition (F.E.R.). Presently F.E.R. [2] is the primary aspect in all sophisticated applications like augmented reality, virtual reality-based systems, customer advertising and marketing, and advanced driver assistant systems. Driver emotion detection based on F.E.R. will become the prominent factor in developing intelligent ADAS for assisting safe driving and ensuring the life security of the people on roads. Even though many research papers have been designed to improve facial expression recognition of drivers for many years, a few challenges are still affecting its performance from further developments such as pose variations, illumination changes, and occlusions [3]. Tracking the driver’s emotion in various angles in different illumination conditions is much needed to predict the correct emotion or behavior of the driver. Occlusions like hair and sunglasses are also significant factors influencing the drivers’ emotions and causing accidents. To prevent these, two novel approaches are proposed to detect the occlusions, illuminations, and pose variations involving driver emotion in which six to seven classes of expressions are being detected from the driver in different illumination conditions. Adding these occlusions, illuminations, and pose invariant-based emotion detection functionalities can enhance the capabilities of the ADAS system for help in maintaining good driving behavior and road safety.

## 2. Related Works

Emotions like happiness, neutral, sadness, disgust, surprise, fear, and anger are usually expressed by a person from his previous and or current performed actions. Some of them are considered negative emotions that influence the driver’s behavior and lead to accidents. These negative emotions trigger the loss of control over driving the vehicle, making the destination unreachable.

Many research developments have been made to monitor the driver’s emotions, thereby assisting the drivers in a smooth and safe driving behavior environment on roads. Different behavioral and physiological signals have been involved and utilized for driver emotion recognition. Using PERCLOS [4] (percentage eye openness tracking), speech [5], face [6], blink [7,8], and body are the source signals to track and predict the driver’s emotion in a behavioral approach. However, physiological signals like E.M.G. [9], E.E.G. [9], E.C.G. [10], E.D.A. [11], PPG&RESP [12], CAN [13], etc., are used to track the driver’s emotion also in a physiological approach. In 2003, Fernandez et al. [14] introduced a driver monitoring system using the speech signal as a source and involving neural networks and SVMs (support vector machines) as a classifier to predict the emotion. In 2007, Grimm et al. [15] proposed a system to predict the driver’s mental state using the speech signal and support vector regression method. Jones and Johnson et al. introduced two different methods using statistical analysis and neural networks with the speech signal as an input source to predict the driver emotion in the years 2008 [16], 2005 [17], and 2007 [18]. Schuller et al. in 2008 [19] introduced an emotion recognition system in an automotive environment using speech signals for emotion tracking. In 2010, Tawari and Trivedhi et al. [20] introduced a speech-based emotion classification framework to predict drivers’ emotions. Boril et al. in 2010 [21], 2011 [22] proposed different driver emotion monitoring systems using G.M.M. (Gaussian mixture models) and SVM (support vector machines) classifiers with a source of a speech signal. In 2012 [23], Alvarez et al. introduced emotional adaptive vehicle user interfaces using logistic model trees, multilayer perceptron, naive Bayes, and logistic regression methods to predict the driver’s emotions using speech signals. In 2011, Tews et al. [24] proposed an emotional human–machine interaction system using a statistical variance method to predict the emotions from the face. Paschero et al. [25] in 2012 introduced a real-time classifier for a vehicle driver’s emotion recognition from the face using the multi-layer perceptron method to classify emotions like happiness, anger, fear, sadness, disgust, and surprise. In the same year, Moriyama et al. [26] introduced a driver emotion recognition system to analyze aggressive moods of the driver from a facial analysis using the mutual subspace method and principal component analysis (P.C.A.).

Agarwal et al. [27] in 2013 introduced an emotion and gesture recognition model for driver assistance with a soft computing tool for human center transportation using a fuzzy rules-based method from the face to predict happiness, surprise, sadness, and anger expressions only. In 2014, Gao et al. [28] proposed an emotion recognition system for driver safety using SVMs to predict anger and disgust expressions. Cruz and Renaldi et al. [29] in 2017 presented an expression analysis summary of motor vehicle operators using CNN (convolutional neural networks) from the driver’s face. Ihme et al. [30] in 2018 proposed a driver emotion recognition from the facial muscle activity using the correlation analysis method. Hoch et al. [31] in 2005 introduced a bimodal fusion of emotional recognition in an automotive environment from both face and speech signals using neural networks and SVMs for speech classification, SVMs for facial classification, and the linear function coefficient fusion method to predict the neutral, positive, and negative expressions of the driver. In 2007, Tischler et al. [32] developed an application of emotion recognition in automotive research using qualitative methods. In 2008, Schuller et al. [33] proposed the detection of security-related effects and behavior in passenger transport using SVMs from face and speech signals of a driver for tracking emotions. In 2012, Boril et al. [34] proposed a multi-model driver’s emotion detection from speech and CAN (controlled area network) signals using the Gaussian mixture mode for speech classification and multiple interval thresholds for the CAN signal analysis. Physiological signals are also used to detect the driver’s emotions. Jeong et al. [35] in 2007 introduced a driver’s emotion index system using a qualitative method. In 2012, Begum et al. [36] proposed a professional driver monitoring system based on a heart rate variability analysis. Later in 2015, Keshan et al. [37] suggested the automobile driver detection system using machine learning approaches. These techniques detect the driver’s emotion from an electrocardiogram (E.C.G.) signal.

Ooi et al. [11] in 2016 proposed a driver emotion recognition framework based on electrodermal activity (E.D.A.) measurements with medical diagnosable physical sensors [38] using SVMs to predict the driver’s emotions. In 2010, Nasoz et al. [39] introduced a driver emotion system using K.N.N. (K-nearest neighbor), Markquardt backpropagation, and resilient backpropagation methods to predict the emotions from E.D.A., E.C.G., RESP (respiratory), and E.M.G. (electromyography) signals. Conzeti et al. [40] in 2012 proposed a bioinspired architecture for on-road emotion monitoring using recurrent neural networks from a photoplethysmogram (P.P.G.) and E.D.A. signals. In 2014, Robodello Mendez et al. [41] developed a body sensor network to detect emotions during the driving environment from E.E.G., E.D.A. using P.C.A., and logistic regression methods. Neska et al. [42] in 2018 proposed a driver emotion system using a random forest approach from physiological functional variable selection signals such as E.M.G., E.C.G., and RESP. Malta et al. [43] in 2011 also analyzed real-world driver’s emotions using the Bayesian network, which combines both behavioral and physiological signals such as E.D.A. and the face. Among all these works, some results [15,25,26,28,31,33] have proposed systems running in a non-car environment, whereas works [20,29,37,40,41,42] have been conducted in a real-time environment. Some results [14,16,17,18,24,30,38,39] have used a simulator environment.

By observing the environmental constraints, the recent driver emotion recognition systems have focused on behavior signals which consume fewer factors that can be considered to design a system with high prediction accuracy.

M. Ali et al. [44] have proposed a multi-inception layer network to address the F.E.R. problem across multiple databases such as CK+ with 93.2% accuracy and FER 2013 with 66.4% accuracy. Ch. Li et al. [45] proposed a multi-network fusion-based CNN with SVM as the classifier and have achieved 70.3% on the FER 2013 dataset. A. Abinav et al. [46] proposed a simple CNN with hyper parameter selectivity and have obtained a 65.7% accuracy on the FER 2013 dataset. M. Riyaz et al. [47] proposed a CNN-based expression network called “Exnet” for F.E.R. and have achieved the better accuracy of 73.5% on the FER 2013 dataset with their model among the pre-trained networks they used. M. Sherwin et al. [48] proposed an F.E.R. system using attentional CNN and have achieved an accuracy of 70% on the FER 2013 dataset. Z. Yuquian et al. [49] proposed a facial expression recognition system using facial action unit feature map selection and sorting in deep CNN and have obtained an accuracy of 88.2% on the KDEF dataset. Y. Liu et al. [50] developed a multi-view face expression recognition using multi-channel pose aware CNNs and have achieved 86.9% accuracy on the KDEF dataset. R. Garcia et al. [51] proposed a deep CNN which can generate a feature vector for expression recognition with the illumination problem and have achieved an accuracy of 95.5% on the KDEF dataset. P. Ramakrishna et al. [52] proposed a real-time neural network with a spatial transformal layer and laplacian operators and have achieved 88.1% accuracy on the KDEF dataset. S. Hari et al. [53] developed a deep learning based F.E.R. system and achieved an accuracy of 96.6% on the KDEF dataset. M. Vijayalakshmi et al. [54] proposed a radial basis function neural network that integrates the shape and texture feature descriptors for expression recognition and achieved an accuracy of 94.2% on the KDEF dataset. B. Hasani et al. [55] proposed a technique to extract temporal relations of consecutive video sequence frames using 3D CNN as well as 3D inception residual network layers to extract spatial relations within facial images using LSTM (long short-term memory). Both the works achieved 93.2% on the CK+ dataset. S. Xie et al. [56] proposed deep comprehensive multi-patches aggregation CNN using hierarchical features and obtained an accuracy of 93.4% on the CK+ dataset. In 2018, Mira Jeong et al. [57] developed a driver facial expression recognition system in real-time for safe driving to monitor the driver’s emotions using a hierarchical, weighted random forest classifier. The authors chose the benchmark datasets CK+ for expression recognition and KMU-FED for an effective real-time driving environment. They achieved an accuracy of 92.2% with a single weighted random forest classifier without hierarchy, 90.9% using a hierarchical, weighted random forest classifier with normal information gain, and 92.6% using a hierarchical, weighted random forest classifier with data similarity for information gain, on the CK+ dataset.

In 2019, M. Patil et al. [58] developed a driver emotion recognition system to enhance human–machine interface in vehicles, trained his model on the CK+ dataset, and achieved 86.7% only. In 2020, M Jeong et al. [59] proposed a lightweight multi-layered random forest model for monitoring driver emotional status, trained his model on the CK+ dataset, achieved 93.4% accuracy, and achieved 95.1% accuracy by training his network on the KMU-FED dataset. In addition, the authors carried out the comparative experiments on the KMU-FED dataset with state-of-the-art models and obtained accuracy such as 89.7% for SqueezeNet [59], 93.8% for MobileNet V2 [59], 94.9% for MobileNetV3 [59], 90.5% for the deep forest, 93.6% for deep random forests, and 91.2% for deep random forests without the backpropagation model.

Although different models have been developed to monitor the human emotions from the captured images with the help of machine learning [60] and deep learning techniques [61], pose variation is also a crucial parameter that should be considered while designing a driver emotion detection system. While designing the models, most of the existing works did not consider this parameter as a significant factor and caused their system to attain less accuracy in driver environmental datasets. To overcome this problem, we have proposed two novel approaches to build an efficient driver emotion detection system, including pose variation conditions, by training our models on the KDEF dataset and achieved remarkable accuracies with the existing F.E.R. methods. We have attained better accuracy on the real-time driving environmental dataset KMU-FED compared with existing driver emotion detection works with this additional functionality.

In this paper, we design and implement a deep convolutional neural network (DCNN) architecture. The design involves a neural network with different optimizers. SGDM supports speed up gradient vectors in the right direction, causing them to converge faster, and the Adam optimizer combines better adaptive gradient and root mean square propagation algorithms. This approach provides better optimization to tackle sparse gradients on noisy conditions and monitor the driver’s emotions in different face rotation angles, occlusions like eyeglasses, hair, and illumination conditions. This system aims to improve the efficiency and performance of the algorithms used. Two novel algorithmic approaches have been developed to monitor and detect emotion to ensure the safety of drivers and vehicles while driving. The proposed algorithms involve preprocessing, segmentation, feature extraction, and classification. The captured images will pass through all stages, and the corresponding class of expression will be detected at the final stage. The performance of the proposed algorithms is compared with the state-of-the-art works using different benchmark datasets and show good performance among them. Two proposed approaches are successfully applied to the real-time virtual driving environment dataset consisting of occlusions and other illumination conditions and obtain better driver expression recognition accuracy.

## 3. Proposed System Methodology

**Figure 1:**
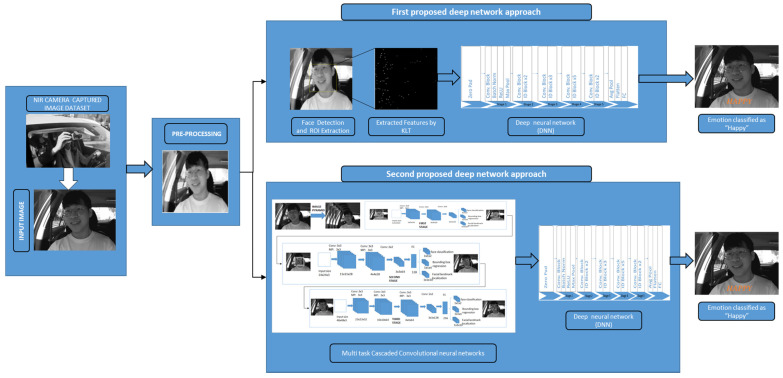
Proposed deep network approaches process flow for driver emotion detection.

In this section, we explain our two proposed approaches for driver emotion detection. The system starts with image acquisition from the datasets, including NIR camera captured real-time driving environment images that are pre-processed for noise removal and image enhancement. In the first approach, the face is detected, and the corresponding ROI (region of interest) is extracted using the Viola–Jones algorithm from the pre-processed image using Haar features. As the KLT (Kanade Lucas Thomasi) algorithm is the fastest among the traditional techniques to monitor the motion estimation and object tracking functions, it has been utilized to track and extract the feature points from the detected face and perform efficiently in real-time processing. The detected ROI from the KLT is given as an input to the deep neural network, which can reduce the neural network’s workload and will enhance the accuracy in classification. In the second approach, we have included the multi-task cascaded convolutional neural networks for more efficient face and landmark detection by passing the pre-processed image through three different neural networks that are cascade-connected. The processed image area is fed to the deep neural network to extract more robust features from them that help in classifying the expressions with high accuracy, as shown in Figure 1. Different optimization and parameter settings were considered while designing and training the two approaches to detect the emotion recognition of the driver, as explained in Section 4.

### 3.1. Pre-Processing

The pre-processing stage of the proposed algorithm involves image resizing, noise removal, and image enhancement. Whenever the image from the dataset is given as input to the preprocessing stage, the image will immediately be resized and sent to the next block for noise removal. The 2D Gaussian and median filters are applied to remove noise from the image in the noise removal stage.

#### 3.1.1. 2D Gaussian Filter

**Figure 2:**
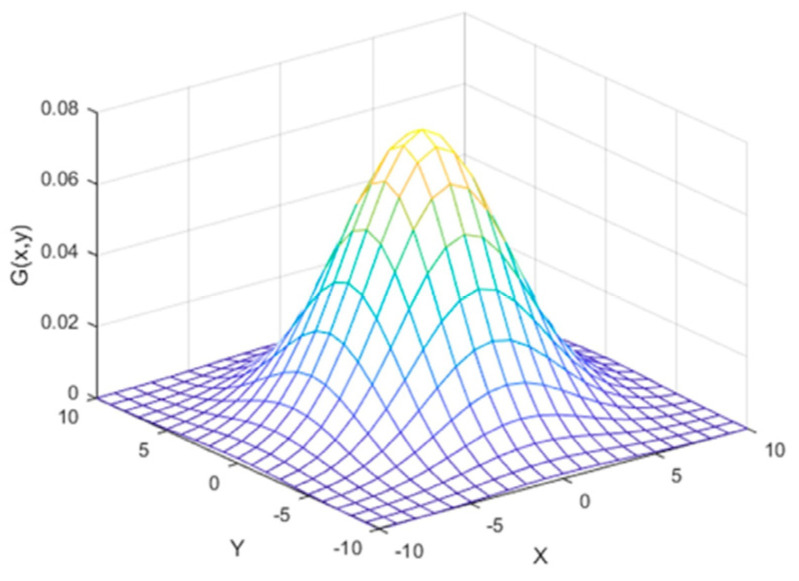
Gaussian distribution.

A smoothing mechanism is used to blur the image using the Gaussian function called Gaussian blur or Gaussian smoothing [62]. Mathematically, blurring an image using the Gaussian function equals convoluting the image with the Gaussian function.

(1) $$G\left(x,y\right)=\frac{1}{2\pi \sigma^{2}}e^{-\frac{x^{2}+y^{2}}{2\sigma^{2}}}$$

where *x*, *y* are the distance coordinates from the origin in the horizontal and vertical axis, respectively, and *σ* is the standard deviation of the Gaussian distribution shown in Figure 2 [63].

#### 3.1.2. Median Filter

It is a nonlinear digital filtering method commonly used to remove the noise from an image by preserving the edges under certain conditions during image pre-processing. It works on replacing each pixel with the median of neighboring pixels. The neighbor pattern is called a window, which slides pixel by pixel over an entire image.

#### 3.1.3. Image Enhancement

This pre-processing block improves the image by increasing the contrast of the low-intensity pixel regions and blurs an image with the smoothening technique.

##### Histogram Equalization

To improve the contrast of an image, histogram equalization [64] is used. This process can be achieved by effectively distributing an image’s most pixel intensity values. This technique increases the image contrast globally by normalizing the histogram of the image. Later wiener filter [65] is applied to remove the blur from the image.

Let us assume the image given as *f*. It is represented as matrix *m_r_* by *m_c_* with pixels intensities 0 to *L* − 1 range, where *L* represents the number of possible intensity values and is mainly equal to 256. The normalized histogram of the image *f* is represented by *p*. Then

(2) $$p_{n}=\frac{\mathrm{number}\;\mathrm{of}\;\mathrm{pixels}\;\mathrm{with}\;\mathrm{intensity}\;n}{\mathrm{total}\;\mathrm{number}\;\mathrm{of}\;\mathrm{pixels}}\;\;\;n=0,1,\ldots ,L-1$$

The histogram equalized image *g* will be defined by

(3) $$g_{i,j}=\mathrm{floor}\left(L-1\right)\sum_{n=0}^{fi,j}P_{n},$$

where floor() rounds down to the nearest integer.

### 3.2. First Proposed Approach for Driver Emotion Detection Using Viola–Jones Face Detection and K.L.T. Feature Tracking with a Deep Neural Network

#### 3.2.1. Face Detection and R.O.I. (Region of Interest) Extraction

Facial image segmentation is crucial in the conventional face expression recognition system. This block is used to detect the face from a pre-processed input image with the help of the Viola–Jones algorithm. The corresponding region of interest is obtained by locating the output’s facial landmarks like the nose, mouth, and eyes. The generated region of interest of an image will be fed to the feature extraction block for further processing in the proposed architecture.

##### Viola–Jones Face Detection Algorithm

It is a prevalent technique for real-time object detection developed by Paul Viola and Michael Jones [66]. There are two stages involved in this algorithm: training and detection. It is developed to detect the frontal faces rather than the faces turned upwards, sideways, and downwards. Before the face is detected, the input image will be converted into grayscale to make the work easier to process with less data. It uses Haar features to search the face inside the box. The box will move from left to right in a tile-wise manner. This algorithm uses three different Haar features to identify the face, namely edge features, line features, and four rectangle features. After detecting the features, the algorithm starts training to identify them by adjusting to a minimum threshold value to classify a feature. Cascade classifiers are used to train the algorithm, and it involves stages. These stages are trained using a boosting technique to train the classifiers at high accuracy to detect a face from an image.

**Figure 3:**
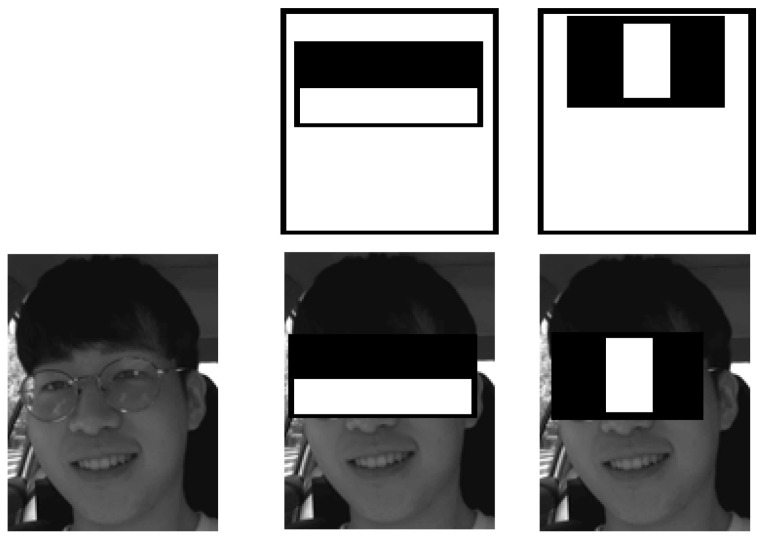
Haar feature face detection.

Two types of Haar feature masks are represented in Figure 3. The extracted window features are in 24 × 24 pixels that slide on the image locations for face detection. Around 162,336 features will be generated from the movement of the scaled Haar masks for the window; many of them are not useful, so the AdaBoost algorithm is used to choose a small number of features by creating a strong classifier with a linear combination of the weak classifier with weights. The weak learner feature f can be represented as

(4) $$h\left(x,f,p,\theta \right)=\{\begin{array}{c} \;1,\;\;if\;pf\left(x\right)<p\theta \\ 0,\;\;otherwise\;\;\;\;\; \end{array}$$

where *x* is 24 × 24 pixel image, *θ* is a threshold, and *p* is a parity.

There are three steps involved in the learning algorithm: training all the weak classifiers and selecting the efficient weak classifier to become a strong classifier component. Then the collected weak classifiers are combined with the other weak learners, which are confirmed as the most efficient in the earlier stage. Next, the weighted error will be calculated to modify the latest weights for the next iteration.

The further weak learners will concentrate on the toughest ones in the training set, and the strong classifier is formed from a combination of the *T* weak classifier with the selection criteria of

(5) $$h\left(x\right)=\{\begin{array}{c} 1\;\;\;if\;\sum_{t=1}^{T}\alpha_{t}h_{t}\left(x\right)\geq \frac{1}{2}\sum_{t=1}^{T}\alpha_{t}\\ 0,\;\;\;\;otherwise\;\;\;\;\;\;\;\;\;\;\;\;\;\;\;\;\;\;\;\;\;\;\;\;\;\;\;\;\; \end{array}$$

(6) $$\;\;\;\;\alpha_{t}=\frac{1}{2}\ln\left(\frac{1-e_{t}}{e_{t}}\right)$$

The weights become larger to a weak classifier with a small amount of error value, and smaller to a weak classifier with a larger error value in classification.

**Figure 4:**
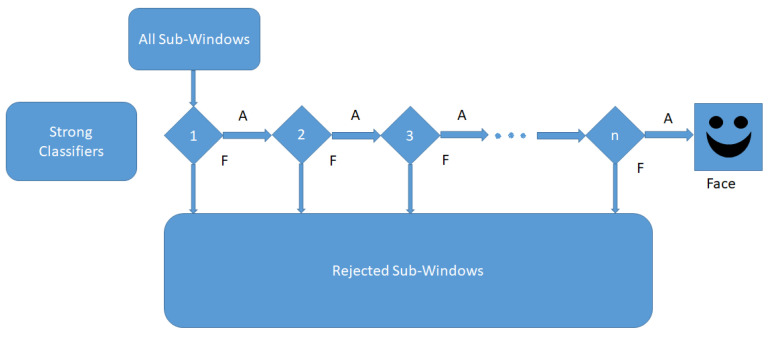
Cascade classifier.

Attention cascade will be created due to the selection of strong classifiers which can obtain a higher detection rate with a smaller classification error. At this cascade configuration shown in Figure 4, the strong classifier will filter and reject the negative windows which contain negative images or non-face images. The configuration will become complex for further stages to achieve a higher detection rate by eliminating negative images with strong classifiers, which will perform higher classifications than previous stages in the cascade structure.

#### 3.2.2. Feature Extraction with K.L.T. (Kanade Lucas Tomasi)

It is one of the best tracking algorithms which is used for feature tracking. The facial detection process requires more computation time. It can cause the detector to fail at the training stage of the classifier when trying to detect the face during continuous motions. Hence, it is necessary to have an optimum algorithm to track the face for features. This algorithm [67] is highly efficient to maintain less computation and a high accurate classifier at the training stage. It works by obtaining feature points [68] through Harris corner detection. The centers of the obtained feature points will help in the tracking process of the facial region. It collects the spatial intensity information to search directly for the best match. This algorithm is based on a gradient weight local search with the approximation of the image second derivative. It involves three steps starting from calculating ‘G’, structure scalar matrix, ‘λ’ pixel cornerness of an image, and maximum cornerness ‘λ_max_’ and maintaining λ > λ_max_ (5–10%). The computation starts from the first partial derivative of image function I(u,v) in vertical and horizontal directions.

(7) $$I_{x}\;(u,v)=\frac{\partial I}{\partial x}\;\left(u,v)\;\mathrm{and}\;I_{y}\;(u,v)=\frac{\partial I}{\partial y}\;(u,v\right)$$

The three computed values for horizontal, vertical, and both direction locations in image (u,v) are:

A(u,v) = I_x_^2^(u,v) (8)

B(u,v) = I_y_^2^(u,v) (9)

C(u,v) = I_x_(u,v) ∗ I_y_(u,v) (10)

These values can form a gradient matrix ‘G’ and are represented as:

(11) $$G=\left[\begin{array}{cc} A & C\\ C & B \end{array}\right]$$

G is a scalar matrix, which means there exist scalars λ_1_ and λ_2_; vectors v_1_, v_2_ such that

A ∗ v_i_ = λ_i_ ∗ v_i_ (12)

The gradient matrix is in sparse matrix form. So, the power method is used to find the largest eigenvalue, and the expected eigenvalue is λ_i_ ≥ 0. The corner point in the image will be given by the maximum eigenvalue.

λ = max(|λ_1_|,|λ_2_|) (13)

#### 3.2.3. Classification

We have opted for the current popular deep neural network, which has achieved good remarks in multi-level class expression classification. This deep neural network is designed to overcome the gradient dissipation by including ReLU as the change in activation function and batch normalization. If the network layers increase, the network can perform effectively in extracting features, though the extraction process is more complicated. Thus, a deeper model yields better results. Another problem is that when the network layers are deep, then the network’s accuracy can be degraded, but the deep neural network [69] is designed to overcome this problem also since the network layers’ depth increases relatively with the magnitude order.

##### Deep Neural Network (DeepNet)

**Figure 5:**
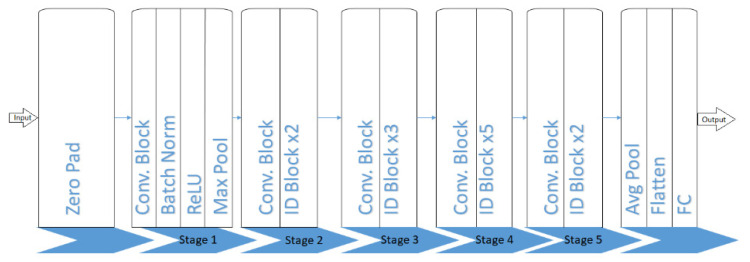
Proposed deep neural network.

Deep neural networks are introduced with a new concept called multi-layers’ deep connections, shown in Figure 5. Using this concept, the number of connections increases, which will increase the computation time of the network, and the accuracy though the layers are very deep.

Zero padding is a method to allow the user to maintain the original size of the input image. It happens during the padding of zeros as a border of pixels around the input image edges. After padding, it will be fed to the convolution layer in which the convolution kernel with its parameters is to be learned. The convolution kernel should be smaller compared to the input image size. The input image is convolved with each kernel to obtain a feature map during the convolution process. The acquired feature map is superimposed with the depth dimension to find the output image at the convolution layer. After receiving the feature map from the convolution layer, the further step is to integrate and classify the features. These features will be fed as an input to the softmax classifier, consuming extensive computation time. To overcome this problem, a pooling layer is used to reduce the feature dimension. It is a process of providing abstractive information. In addition, it reduces parameters, features, and the matrix size, which is generated by the convolution layer to simplify the complexity in calculation at the convolution layer. Two main operations are performed, namely average pooling and max pooling. The average pooling usually reduces the increased variance due to neighborhood size and restores the background data information of an image. Average pooling also guarantees the integrity of the information transmitted and decreases parameter dimensions. Max pooling chooses features with good classification for easy recognition and nonlinear characteristics. In addition, max pooling reduces the parametric error in the convolution layer, which creates a change in the calculated mean value. For instance, if the feature to identify is a car, then until there is a car in any part of the area of an image, it will consider there is a car in the whole image.

The batch normalization layer is an optimization method used in the training phase. A batch is the set of images that are trained on the network. The purpose of normalization is for the input data to make the neural network learning process to learn the data distribution. After the training data distribution has differed from the testing data, the generalization capability of the network will be degraded, which in turn affects the training speed of the network due to another factor of training different batches of data with various distributions. Selecting different parameters such as parameter initialization, learning rate, and weight attenuation coefficient, etc., manually will consume most of the time. To adjust these parameters, automatically updated, batch normalization is used. The main principle of this batch normalization layer is to perform a normalization process for each layer acting as input, and a separate normalization layer is added to each layer. The rectified linear unit, in short ReLU, is the activation function used to append the nonlinear factors and make a nonlinear combination of inputs to make the deep neural network classification capability stronger. A piece-wise function makes all negative values zero and does not change all the positive values. For instance, if the input value is negative, the output will be zero. The neuron will not get activated, which means only a few neurons will get activated with the positive values, making the network efficient in computation.

### 3.3. Proposed Algorithm for Driver Emotion Detection Using a Multi-Task Cascaded Convolutional Neural Network with a Deep Neural Network

We have used the same pre-processing blocks in the second proposed algorithm and used multi-task cascaded convolutional neural networks for face and facial landmark detection.

#### 3.3.1. Multi-Task Cascaded Convolutional Neural Networks

**Figure 6:**
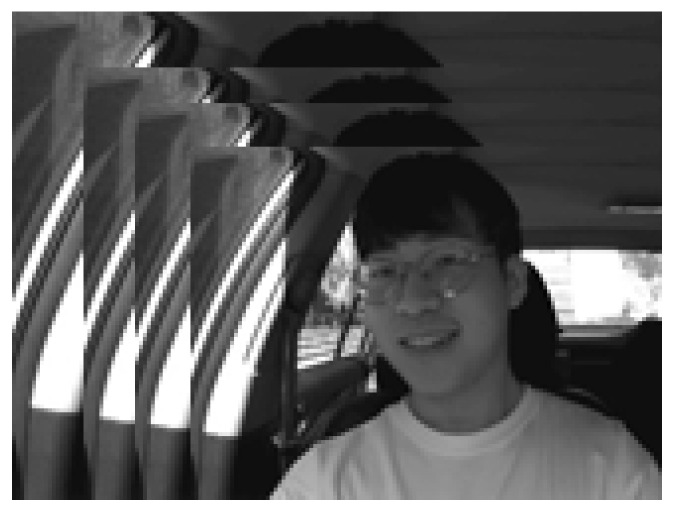
Image pyramid.

The critical stages in the conventional face expression recognition system are face detection, feature extraction, and expression classification. The main stage is feature extraction, which influences the network’s accuracy. The traditional convolutional neural network architecture gives a better classification with some challenges like pose variations and occlusions affecting the accuracy of the network. To improve the accuracy of the networks, multi-task cascaded convolutional neural networks [70] are used for face detection and facial landmark detection. There are three neural networks present in the multi-task cascaded neural networks, which are cascaded in three stages. The first stage utilizes a shallow convolutional neural network to rapidly generate candidate windows. The second stage filters the generated candidate windows to pass through a complex convolutional neural network. At the third stage, the third convolutional neural network, which is more complex than the previous two networks, will be used to filter further to identify the facial landmark locations. Before proceeding with the input image to the three-staged cascaded neural network, the input image needs to be scaled in different sizes to construct an image pyramid, as shown in Figure 6.

##### First Stage

**Figure 7:**
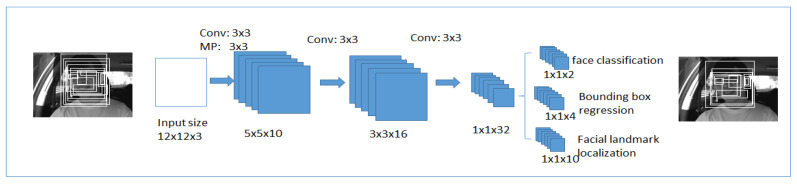
The first stage in multi-task cascaded convolutional neural networks.

The first network is a fully convolutional neural network shown in Figure 7, will be used to produce the candidate window and border regression vectors. Bounding box regression [71] is a reliable method to predict the box localization if the target detects an object of a class that was already defined. The overlapped regions are combined after the bounding boxes are determined. At the final output, candidate windows will reduce the size of the candidates’ volume.

##### Second Stage

**Figure 8:**
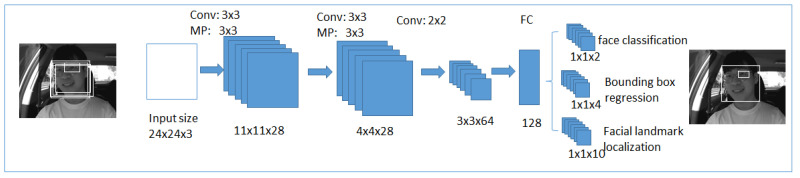
The second stage in multi-task cascaded convolutional neural networks.

The obtained candidates from the first stage will be given to refining the convolutional neural network, shown in Figure 8. In this network, the fully connected layer is present at the output stage of the architecture. This refines the convolutional neural network, further filters candidates, applies calibration on bounding box regression, and uses non-maximum suppression to combine the overlapped candidates. It generates a four-element bounding box created for face detection, and a ten-element bounding box vector created for facial landmark localization.

##### Third Stage

**Figure 9:**
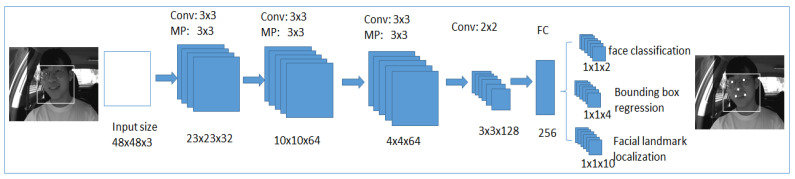
The third stage in multi-task cascaded convolutional neural networks.

It is the last stage in which the output neural network is used shown in Figure 9. The output network performs as the same as a refined neural network in the second stage. It filters the candidates further to provide more details about the face, and five positions related to five facial landmarks will be detected.

Every neural network in multi-task cascaded convolutional neural networks will generate three parts at their stages, and the corresponding loss will also have three parts. Cross-entropy loss function is used directly for detecting faces from an image:

(14) $$L_{i}^{det}=-\left(y_{i}^{det}\log\left(p_{i}\right)+\left(\;1-y_{i}^{det}\;\right)\left(1-\log\left(p_{i}\right)\right)\right)$$

where $p_{i}$ is the input image probability and det is the original label in $y_{i}^{det}$.

Common Euclidian distance is used to find the loss function for boundary box regression and can be calculated as:

(15) $$L_{i}^{det}={{\left|\left|{\hat{y}}_{i}^{box}-y_{i}^{box}\right|\right|}_{2}}^{2}\;$$

The network predicted coordinate is ${\hat{y}}_{i}^{box}$ and the coordinate of actual real background is $y_{i}^{box}$.

Key point decision loss function:

(16) $$L_{i}^{landmark}\;=\;{{\left|\left|{\hat{y}}_{i}^{landmark}-y_{i}^{landmark}\right|\right|}_{2}}^{2}$$

Here in the above equation, the network predicted coordinate is ${\hat{y}}_{i}^{landmark}$ and the coordinate of the actual real key point is $y_{i}^{landmark}$. The final total loss is formed by adding the three losses that are multiplied by their weights.

This algorithm improved the classification accuracy by utilizing MTCNN for face detection and facial landmarks extraction. Later, the obtained will be given to DeepNet for classification to predict the driver’s emotion.

This paper proposes two novel deep network approaches to detect driver’s emotions. The first approach utilizes the Viola–Jones face detection method for frontal face and different angular faces’ detection. Viola–Jones face detection is limited to frontal face detection and obtaining facial landmarks. The corresponding facial features are being detected and tracked by the Kanade Lucas Tomasi method, which are given to the deep neural network for emotion detection. Connecting these methods to a deep neural network brings higher accuracy in detecting a driver’s emotions in different angular rotations of the face and various illumination condition scenarios with partial occlusions like sunglasses and hair involved. The achieved accuracy improves with the second novel deep network approach. We use multi-task cascaded neural networks for face detection and alignment with facial landmark detection in various angular rotations. The obtained features are trained and classified using a deep neural network with ReLU modified to a combination of batch normalization and leaky ReLU to avoid the occurrence of a dying ReLU problem at ReLU, which causes some neurons to die during training. Due to this problem, stochastic gradient descent (S.G.D.) optimization cannot affect the network with the property of the gradient becoming zero even though the input value is negative. So, to overcome this, we use the Adam optimizer, which is faster, stable, and converges faster than S.G.D.

## 4. Experimental Results

Various databases are introduced to evaluate the facial expression recognition performance from image sequences and are used for developing different applications. From those databases, we have chosen the most used and popular benchmark datasets for facial expression recognition, such as the extended Cohn Kanade database (CK+) [72], facial expression recognition (FER) 2013 [73], and Karolinska directed emotional face (KDEF) [74]. As we focused on driver emotion detection, we also have chosen the KMU-FED [75] database in which driver face expression recognitions are captured in a real-time driving environment. Firstly, we explained the databases utilized for performance evaluation and further explained the obtained results by comparing them with the state-of-the-art methods. We have considered the model’s true positive and true negative outcomes for the performance evaluation metrics’ calculation. This driving emotion detection model is developed using MATLAB in a system environment that includes an Intel 9th Generation i5-9300H Quad-Core Processor with 12 G.B. of RAM in the Windows 10 operating system and executed on a NVIDIA GeForce GTX 1650 GPU.

The parameter settings for the training of our two proposed approaches on all four databases are shown in Table 1. For the first approach, we have chosen a stochastic gradient descent optimizer with momentum having a learning rate of 0.01 for the first proposed approach and an Adam optimizer with a learning rate of 0.001 for the second proposed approach with cross-entropy as loss function, ReLU as the activation functions in both of them, and trained to a maximum number of 100 epochs.

### 4.1. Databases

(I) CK+

**Figure 10:**
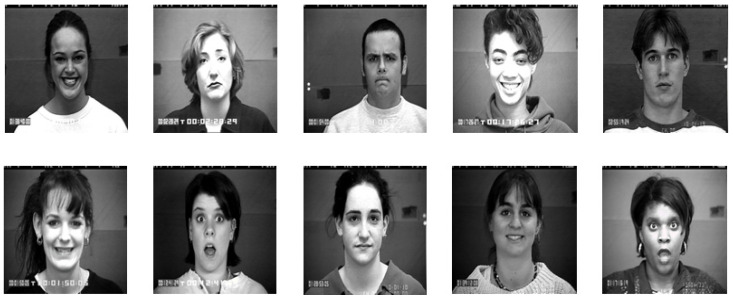
Sample images from CK+ database.

It is known as the “Extended Cohn-Kanade Database” [72], one of the most widely used databases for evaluating face expression recognition systems. It is an extensively utilized facial expression database provided in a laboratory-controlled environment. This database shown in Figure 10 contains 593 image sequences from a total number of 123 subjects in the age range from 18 to 50 years, including a variety of genders and origins of 81% Euro-American, 13% Afro-American, and the remaining 6% are others. The involved images have a facial shift from a neutral expression to the targeted peak expression with a pixel resolution of 640 × 480 and 640 × 490 in grayscale.

(II) FER 2013

**Figure 11:**
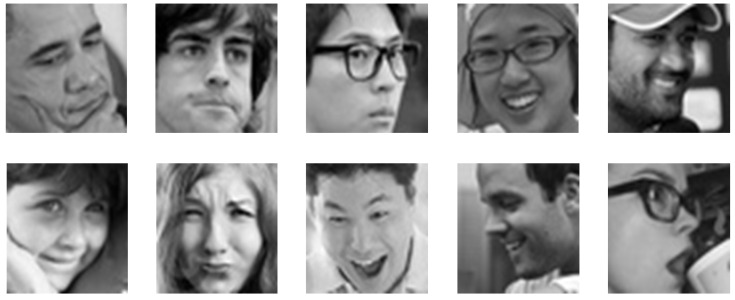
Sample images from FER 2013 database.

The facial expression recognition 2013 (FER 2013) [73] database shown in Figure 11 was introduced in ICML Challenges in representation learning in 2013. It includes different images captured in a wild environment and created using Google image search API (application program interface), and the corresponding faces are automatically registered. It contains 35,887 images with a pixel resolution of 48 × 48. These images have more variations such as facial occlusions with hand, partial occlusions like hair, eyeglasses, and images in low illumination conditions, and face angular rotations.

(III) KDEF

It is known as the Karolinska directed emotional face (KDEF) [74] database, consisting of 4900 static face images captured from 35 female and 35 male subjects. Each subject’s facial expression is captured twice in five different face angles of −90°, −45°, 0°, 45°, and 90°, which results in 980 image sets for each angle. This database was initially developed for psychological and medical research purposes in Sweden, but currently, it is the most suitable dataset for the performance evaluation of emotion recognition experiments.

(IV) KMU-FED

**Figure 12:**
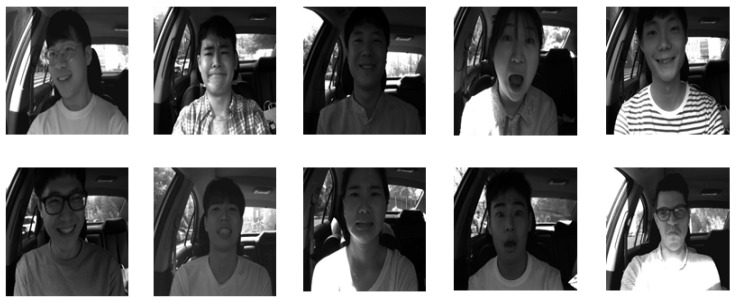
Sample images from KMU-FED database.

To evaluate and verify the performance of our proposed approaches for driver emotion detection in a real-time driving environment, we have selected the Keimyung University facial expression of drivers (KMU-FED) [75] database. KMU-FED contains the captured facial expression images in an actual driving environment, shown in Figure 12. These drivers’ facial expression images in the dataset are captured using the NIR (near-infrared) camera installed on the dashboard or the steering wheel. Twelve subjects were involved and generated 55 image sequences with different illumination condition variations like front light, left light, right light, and back light with partial occlusions like hair or eyeglasses. The images have a pixel resolution of 1600 × 1200.

### 4.2. Performance Evaluation

#### 4.2.1. Experiments on CK+ Database

In order to verify the effectiveness of the two proposed approaches for driver emotion detection, firstly we have compared their performance with the state-of-the-art approaches which have used the CK+ database to develop face expression recognition systems earlier: (1) D.N.N. utilizing multi-inception layers [44]; (2) the inception-resnet network which used LSTM to enhance the 2D inception-resnet module [55]; (3) single weighted random forest without hierarchy [57]; (4) hierarchical weighted random forest with normal information gain [57]; (5) hierarchical weighted random forest with data similarity for information gain [57]; (6) facial expression recognition using hierarchical features with deep comprehensive multi-patches aggregation convolutional neural networks [56]; (7) using lightweight multi-layer random forests for driver emotion monitoring [59]; (8) first proposed deep network approach using Viola–Jones face detection and Kanade Lucas Thomasi feature extraction with deep neural network; (9) second proposed deep network approach using multi-task cascaded neural networks and deep neural network represented in Table 2 and in which it shows the better accuracies that are achieved using deep neural networks and recurrent neural networks. However, using classification algorithms like random forests with weights, hierarchy, and lightweight multi-layered random forests have achieved a maximum accuracy of 93.4% which is achieved with our first proposed approach and the accuracy is improved to 96.1% with our second proposed approach which is 5.2% greater than the accuracies obtained by the state-of-the-art methods on the CK+ database.

#### 4.2.2. Experiments on the FER 2013 Database

To evaluate the performance of the proposed two approaches, we have compared the recognition accuracy of the proposed approaches with the seven state-of-the-art approaches which use either deep neural networks or conventional machine learning algorithms: (1) a D.N.N. that utilizes various inception layers [44]; (2) a convolutional neural network which involves multi-network fusion [45]; (3) simple CNN models to evaluate the effects on accuracy due to kernel size and filters [46]; (4)–(6) an efficient approach called eXnet [47] for emotion recognition in the wild; (7) an attentional convolutional neural network called deep-emotion [48]; (8) the proposed first deep network approach using Viola–Jones face detection and Kanade Lucas Tomasi feature extraction with a deep neural network, and the second proposed deep network approach using multi-task cascaded neural networks and deep neural networks with different optimizers involved.

From Table 3, implementing facial expression recognition using deep neural networks [44] in which the network’s depth is increased by adding inception layers has obtained accuracy which is 17.2–18.1% less than our proposed approaches. However, CNN- M.N.F. [45] is a multi-network fusion from Tang’s network in which support vector machine classifier and Caffe-ImageNet, a deep level based neural network, have implemented but achieved an accuracy of 13.3–14.2% less than that of our proposed approaches. Simple CNN models [46] with hyperparameter selectivity for improved accuracy have achieved 17.83–18.73% less than our proposed approaches. An efficient convolutional neural shallow network architecture called Expression Net [47] that reduced the layers for fast performance achieved 10.1–11% less accuracy than that of our proposed approaches. The authors trained the pre-trained networks eXnet-Resnet [47], eXnet-DeXpression [47] with the same parameters in which the expression network eXnet was built earlier and achieved 13.4–16.5% less accuracy when compared to our proposed approaches and deep-emotion [48]. Our network is created using an attentional convolutional neural network to apply attention to special regions, which are crucial for facial expression detection, and has achieved 13.6–14.5% less accuracy than our first and second proposed approaches. From the comparison, we observed that our proposed approaches obtained better accuracies than the works that utilized deep neural networks with machine learning methods involved and a fusion of multi-networks and feature-oriented attentional neural networks.

#### 4.2.3. Experiments on KDEF Database

For performance evaluation, we have compared our two proposed approaches with the state-of-the-art methods that used the KDEF database earlier in Table 4: (1)–(2) a deep convolutional neural network AlexNet which is pre-trained before taken, and classification is completed with the proposed two feature selection schemes to choose either the selection of facial action units by training with binary action unit detectors for every feature map and sort them [49] or detecting the feature maps in the areas inside the face areas found by the deconvolutional neural network [49] and this selection of feature maps are influencing the classification robustness, but both these schemes achieved accuracy which is 10.2–12% less than that of our first proposed approach, and 10.9–12.7% less than our second proposed approach; (3) a multi-view facial expression is recognized by multi-channel pose aware convolutional neural network [50] and has achieved accuracy which is 11.5–12.2% less than that of our proposed approaches; (4) a CNN [51] which is pre-trained with deep stacked convolutional auto encoder (DSCAE) which will generate a feature vector for expression recognition by overcoming the illumination problem and has achieved better accuracy compared to the other five state-of-the-art methods but still 2.9–3.6% less than that of our proposed approaches; (5) adding the gradient and laplacian inputs to an image given to CNN [52] helps in recognizing the facial expression but with accuracy which is 10.2–10.9% less than that of our proposed first and second approaches; (6) a usage of the Haar classifier before feeding into the deep neural network can reduce convergence time more than others without having it and it achieved the best accuracy compared to the other state-of-the-art methods but still 1.8–2.5% less than that of our first and second proposed approaches [53]; (7) a radial basis function neural network [54] which uses a feature integration of shape descriptors and texture features for expression recognition has achieved accuracy which is 9.6–10.3% less than that of our first and second proposed approaches.

#### 4.2.4. Experiments on KMU-FED Database

For evaluating our proposed approaches in a real-time driving environment, we have compared with the seven state-of-the-art methods in Table 5: (1) hierarchical weighted random forest classifier with the geometrical feature vectors generated from facial landmarks are used to classify the facial expressions from an input image in the real-time driving environment and achieve the accuracy which is 4.1–5.0% less than that of our proposed first and second approaches [57]; (2) a connected convolutional neural network [76] which consumes both low level and high level features has achieved a better accuracy in seven state-of-the-art methods but still 0.8–1.7% less than that of our proposed first and second approaches’ accuracy; (3)–(6) to know the performance evaluation of KMU-FED database with deep neural networks SqueezeNet [59], MobileNetV2 [59], MobileNetV3 [59] which are pre-trained earlier are taken to train with KMU-FED database and achieve an accuracy that is 8.4–9.3%, 4.3–5.2%, and 3.2–4.1% lower than our first and second approaches, respectively, a light weight multi-layered random forest [59] classification model involving the combination of angle and distance ratio feature vectors which does not involve any deep neural network has achieved an accuracy that is 3–3.9% lower than our proposed deep network approaches; (7) a pre-trained deep convolutional neural network, VGG16 [77] taken and trained with driving dataset with different angles and illumination differences achieves an accuracy that is 3.9–4.8% less than our novel proposed deep network approaches. By comparing with all the state-of-the-art methods, our proposed approaches have achieved better accuracy than the existing works. However, they have used machine learning-based classification models or deep convolutional neural network models.

### 4.3. Emotion Recognition Results

**Figure 13:**
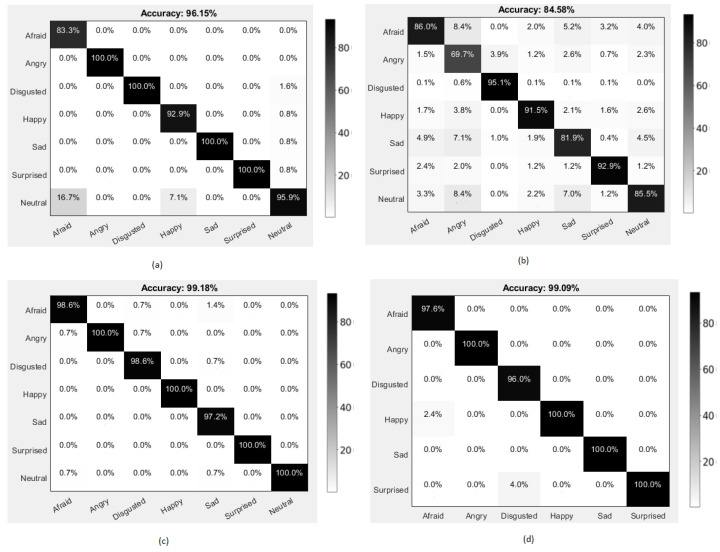
Confusion matrices with accuracy (%) of the second proposed approach using different databases (**a**) CK+ database, (**b**) F.E.R. 2013 database, (**c**) KDEF database, and (**d**) KMU-FED database.

In the process of evaluating the performance of our classification model, we constructed the confusion matrices of our high accuracy obtained proposed approach for the CK+, FER 2013, KDEF, and KMU-FED databases, respectively, as represented in Figure 13. The first figure, i.e., Figure 13a, shows that angry, disgusted, sad, and surprised expressions were classified with high accuracy. In contrast, the afraid expression was classified with significantly less accuracy compared to the other expressions for the CK+ database. Figure 13b represents the confusion matrix for the FER 2013 database, a wild database involving most of the possible challenges affecting the facial expression classification. The disgusted expression was classified with high accuracy, and the angry expression was classified with low accuracy compared to the remaining expressions in the database. In Figure 13c, angry, happy, surprised, and neutral expressions were highly accurate, whereas the sad expression was classified with low accuracy in the KDEF database. Lastly, Figure 13d represents the KMU-FED database in which the images were captured in a real-time driving environment with different illumination changes and partial occlusions involved while driving. The expressions angry, happy, sad, and surprised were classified with high accuracy, and disgusted was classified with lesser accuracy than the expressions in the database. The highest accuracy in classifying the four expressions, namely angry, which causes aggressive driving, happy which causes the anxiety in driving behavior, sad which can influence negative driving behavior, and surprised which can intensify the emotional level of driving can show that our deep network approaches are well suitable to be used in developing an automobile surveillance system application [78] to monitor these crucial emotions of a driver, as our proposed deep network approaches are focused on driver emotion detection.

## 5. Conclusions

This paper proposes novel deep network approaches to determine the driver’s emotions in a real-time driving environment through facial expression recognition to assist advanced driver assistance systems in intelligent vehicles. These deep network approaches are evolved by detecting the face from captured images by Viola–Jones, and the corresponding features are tracked using the Kanade Lucas Tomasi method. The faces are then fed to the deep neural network for classification and recognition as described in the first proposed approach. In contrast, the second approach has used multi-task cascaded convolutional neural networks for face detection alignment and tracking the features which are given to the deep neural network. These two approaches are trained with different optimizers on the selected benchmark datasets. The work presented in this paper achieves the state-of-the-art result to solve the problems of emotions reflecting a driver’s behavior such as the changes in illumination, side angle positions of the sunlight, occlusions like hair and sunglasses, and different angular face rotations. To assess our proposed approaches’ detection capability, we have conducted experiments on four benchmark databases CK+, FER 2013, KDEF, and KMU-FED, which address the above-mentioned challenges.

Due to the COVID-19 pandemic, another challenge has been raised for face emotion detection because of face masks and face coverage. This problem has been discussed in other works [79,80,81,82] addressing the challenge. However, none of them include machine learning techniques to solve the problem. This study can be extended for future work by proposing computer vision and machine learning approaches for detecting the driver’s facial emotions from the masked faces. Moreover, extra experiments can be investigated to improve and utilize more state-of-the-art techniques to match the runtime requirements of this application.

## Figures and Tables

**Table 1:** Parameter settings used to train our deep network approaches on all four databases.

Databases	Parameters	First Approach-Values	Second Approach-Values
	Image Size	256 × 256	256 × 256
	Optimizer	Stochastic Gradient Descent (S.G.D.)	Adam
CK+	Loss Function	Cross-Entropy	Cross-Entropy
FER 2013	Activation Function	ReLU	ReLU
KDEF	Batch Size	128	128
KMU-FED	Learning Rate	0.01	0.001
	Epochs	100	100
	Momentum	0.9	0.9
	Validation Frequency	30	30

**Table 2:** Comparison of proposed approaches with the state-of-the-art methods on CK+ database.

Comparison Methods	Accuracy (%)
DNN [44]	93.2
Inception-Resnet and LSTM [55]	93.2
Single-WRF [57]	92.2
Hierarchical W.R.F. with Normal Information Gain [57]	90.9
Hierarchical W.R.F. with Data Similarity [57]	92.6
DCMA-CNN [56]	93.4
LMRF [59]	93.4
First Proposed Approach	93.4
Second Proposed Approach	96.1

Performance accuracies of different methods adapted from different papers.

**Table 3:** Comparison of proposed approaches with the state-of-the-art methods on FER 2013 database.

Comparison Methods	Accuracy (%)
D.N.N. [44]	66.4
CNN-MNF [45]	70.3
Simple CNN Model [46]	65.7
eXnet [47]	73.5
eXnet-Resnet [47]	71.1
eXnet-DeXpression [47]	68.0
Deep-Emotion [48]	70.0
First Proposed Approach	83.6
Second Proposed Approach	84.5

Performance accuracies of different methods adapted from different papers.

**Table 4:** Comparison of proposed approaches with the state-of-the-art methods on KDEF database.

Comparison Methods	Accuracy (%)
TLCNN [49]	86.4
TLCNN-FOS [49]	88.2
MPCNN [50]	86.9
DSCAE-CNN [51]	95.5
STL + GRADIENT + LAPLACIAN RTCNN [52]	88.1
DL-FER [53]	96.6
RBFNN [54]	88.8
First Proposed Approach	98.4
Second Proposed Approach	99.1

Performance accuracies of different methods adapted from different papers.

**Table 5:** Comparison of proposed approaches with the state-of-the-art methods on KMU-FED database.

Comparison Methods	Accuracy (%)
Facial Landmarks + WRF [57]	94.0
CNN [76]	97.3
SqueezeNet [59]	89.7
MobileNetV2 [59]	93.8
MobileNetV3 [59]	94.9
LMRF [59]	95.1
VGG16 [77]	94.2
First Proposed Approach	98.1
Second Proposed Approach	99.0

Performance accuracies of different methods adapted from different papers.

## Data Availability

Not applicable.
